# A call for communication, compassion and care

**DOI:** 10.4102/sajpsychiatry.v30i0.2369

**Published:** 2024-09-26

**Authors:** Marenet Jordaan

**Affiliations:** 1 Department of Journalism, Faculty of Arts and Social Sciences, Stellenbosch University, Stellenbosch, South Africa

I really thought she was Satan.

Now that I think about it – more than 15 years later – I realise it must have been just as traumatic for my psychiatrist as it was for me. In that moment, however, she personified my deepest fears. That is why I broke her spectacles in two, a last-ditch psychotic attempt at warding off Evil. The result of this unfortunate attack was an instantaneous injection after which things for me immediately went dark.

People who have never seen me in this state find it difficult to make sense of this anecdote. I am generally a peace-loving, docile person. That is: except when I am manic beyond belief. I have learnt that beliefs, in fact, had a lot to do with my psychotic episodes. The ancient myths of Good versus Evil and all that. Maybe I am lucky not to *see* such evil imagery. No scary horns or fires and flames for me. Yet, *feeling* such an evil presence was just as horrific, as is recalling those visceral feelings now. When I think of that moment with the ‘devil’, my throat sometimes still closes with angst and fear.

I realise now that these perceptions are not fair to the doctor in question. Our relationship was generally okay – albeit mainly centred on medication. I could perhaps critique her bedside manner during my 3-week long stay in the (private) clinic that followed on my psychotic episode. Suffice it to say, once I was discharged, I found someone else with whom to continue treatment.

My second manic episode was around 5 years later. The intense experience of Good versus Evil resurfaced, but this time there were a few moments of lucidity during the psychosis. So much so that I have a vivid memory of the young psychiatrist, working on a public holiday at the same clinic, sitting me down to say that if I do not calm down and allow him to inject me, I would be moved to a state-run facility. Despite the injection that followed, this memory is substantially more positive than my previous experience. I remember an atmosphere of calm and care.

I have compassion for my former doctor. I cannot imagine how hard it was to be on the receiving side of an attack by a combative, psychotic, bipolar patient. Nothing is pretty about that picture. Trust me, I know. Those visuals seem to be forever imprinted on my mind.

Yet, perhaps there was a need for more engagement about what to do after the fact. After the injection. After the days of dreamless sleep. After waking up in a ward where the bathroom doors could not lock. After being confronted with one’s own demons – literally.

I do not blame my former doctor. Yet, I do wish she could have better understood (from my perspective) the sense of utter failure and incredible shame that accompanied having been so completely out of control. Do not tell me it was not ‘me’. Do not tell me that it was my ‘subconscious’ acting out. Do not tell me that I had no control over it. Until you have been in a body with a brain that malfunctions so spectacularly, you have very little right to try and appease me in this way. Let us rather find a way *through* such an experience – together.

I realise that the talking-about-it bits of treating a psychiatric illness are usually reserved for clinical psychologists and therapists in various guises. I would advocate, however, for a world in which patients’ time spent with *all* psychiatrists involves more than just a check-in about the list of medications. The pills might work, but the memories remain.

M.J. (PhD Journalism), living with diagnosed bipolar mood disorder since 2001.

Thank you to all psychiatrists who have been part of my journey.

The views and experiences expressed in this article are reflective of a personal journey and should not be read as a piece of academic work (despite the author being an academic).

